# Fabry disease with acute myocardial infarction, left ventricular thrombosis, and pericardial effusion: A case report

**DOI:** 10.1097/MD.0000000000029427

**Published:** 2022-05-27

**Authors:** Shanshan Zhou, Xiaocong Wang, Hui Xu, Jing Li, Liping Zhang, Hang Li

**Affiliations:** The Center of Cardiovascular Diseases, The First Hospital of Jilin University, Changchun, China.

**Keywords:** alpha-galactosidase, cardiac resynchronization therapy with an implanted defibrillator, Fabry disease, X chromosome

## Abstract

**Rationale:**

Fabry disease (FD) is a rare, X-linked lysosomal deposition disease characterized by multi-system symptoms. The accumulation of globotriaosylceramide in various organs, such as the kidneys and heart, as well as the nervous system, has been speculated to be the mechanism involved in tissue damage, including vascular impairment with thrombotic events.

**Patient concerns:**

Here, we describe a 72-year-old male patient diagnosed with FD, who first presented with acute myocardial infarction, left ventricular thrombosis, and pericardial effusion, accompanied by cardiac hypertrophy.

**Diagnoses:**

A physical examination showed that he was hemodynamically stable and an electrocardiogram showed ventricular tachycardia (Fig. 1A). The single obvious abnormality was an ST segment depression with a preterminal negative T wave in leads I and aVL (Fig. 1B). Coronary angiography revealed regular findings (Fig. 2). Echocardiogram conducted at our hospital revealed hypertrophy, ejection fraction 40%, pericardial effusion (Fig. 3). Speckle tracking two-dimensional echocardiography strain analysis technology confirmed left ventricular thrombosis, and also revealed decreased movement of the inferior and posterior walls, the basal segment of the posterior wall was locally fibrotic (Fig. 4A and B). Further, myocardial contrast echocardiography confirmed left ventricular thrombosis (Fig. 4C). Cardiovascular magnetic resonance imaging indicated biventricular uneven hypertrophy, which was considered metabolic cardiomyopathy, with diffuse fibrosis of biventricular walls, apical thrombosis, and ischemic cardiomyopathy in the basal segment of the left ventricular lateral wall and left ventricular anterior wall (Fig. 5). Serum alpha-galactosidase concentration was 0.7 nmol/h/mgPr (normal range, 29.0–64.4 nmol/h/mgPr). Subsequent genetic testing revealed that he was hemizygous for a previously reported missense mutation (c.902G>A) inexon 6 of the *GLA* gene,^[1]^ which induce p.R301Q (p.Arg301Gln), confirming a diagnosis of FD (Fig. 6).

**Interventions:**

Orally administered drugs included rivaroxaban, sacubitril valsartan, beta blockers, dapagliflozin, and mineralocorticoid receptor antagonist. Cardiac resynchronization therapy with an implanted defibrillator was implemented to prevent sudden death.

**Outcomes:**

At present, he is still in follow-up and there have been no adverse events.

**Conclusion:**

Our case suggests that clinicians should consider the possibility of FD in patients with acute myocardial infarction and cardiomyopathy. A detailed analysis of subtle historical clues would help promote earlier diagnosis of FD.

## 1 Introduction

Fabry disease (FD) is an X-chromosome-linked inherited disorder of glycosphingolipid metabolism caused by deficient or absent lysosomal alpha galactosidase A activity, which results in progressive build-up of globotriaosylceramide in the cells of various tissues.^[1,2]^ The severity of FD also depends on sex, with males being more severely affected than females. FD presents with multisystemic signs and symptoms, including angiokeratoma, acroparesthesia, diaphoresis abnormalities, cornea verticillata, and chronic or episodic pain, as well as cardiovascular, cerebrovascular, and renal disorders, such as cardiomyopathy, arrhythmia, stroke, and proteinuria, resulting in limited life expectancy.^[2]^ In the heart, glycosphingolipide position causes progressive left ventricular hypertrophy that mimics the morphological and clinical characteristics of hypertrophic cardiomyopathy.^[3,4]^ Here we report a case of an older male patient with FD, acute myocardial infarction, left ventricular thrombosis, and pericardial effusion.

## 2 Case presentation

**Figure 1:**
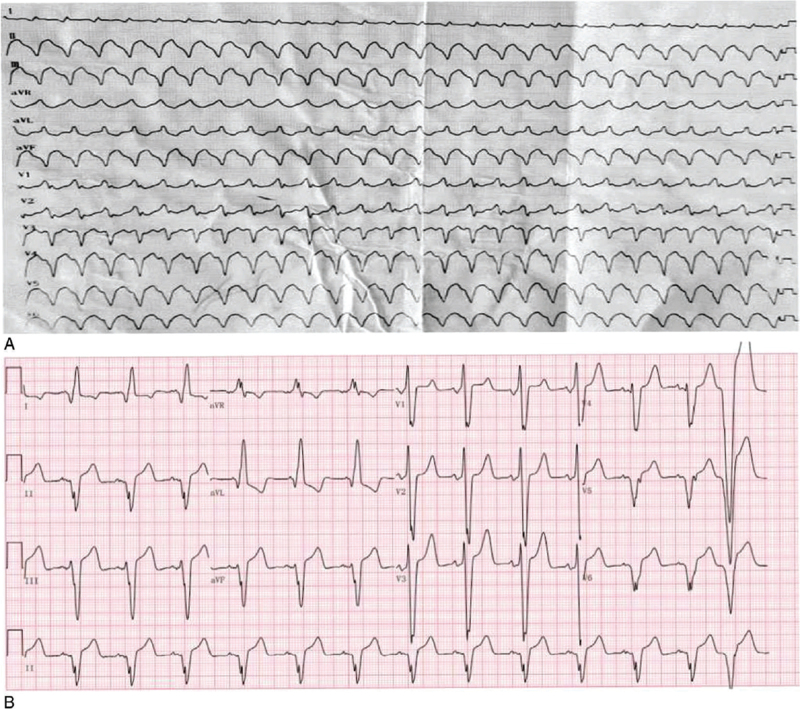
(A) ECG: ventricular tachycardia. (B) ECG after intravenous amiodarone: sinus rhythm, 72/min, left axis deviation, left anterior branch block, and intraventricular block. ST segment depression with a preterminal negative T wave in leads I and aVL. ECG = electrocardiogram.

**Figure 2:**
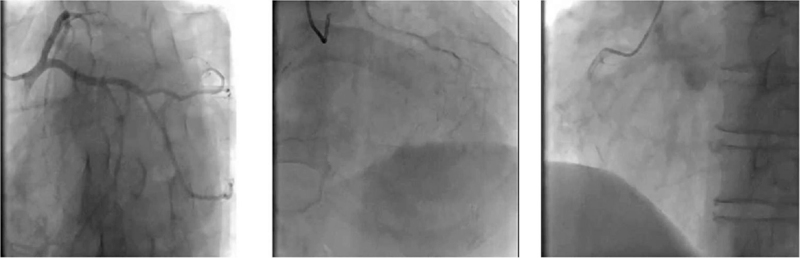
Coronary angiography revealed regular findings.

**Figure 3:**
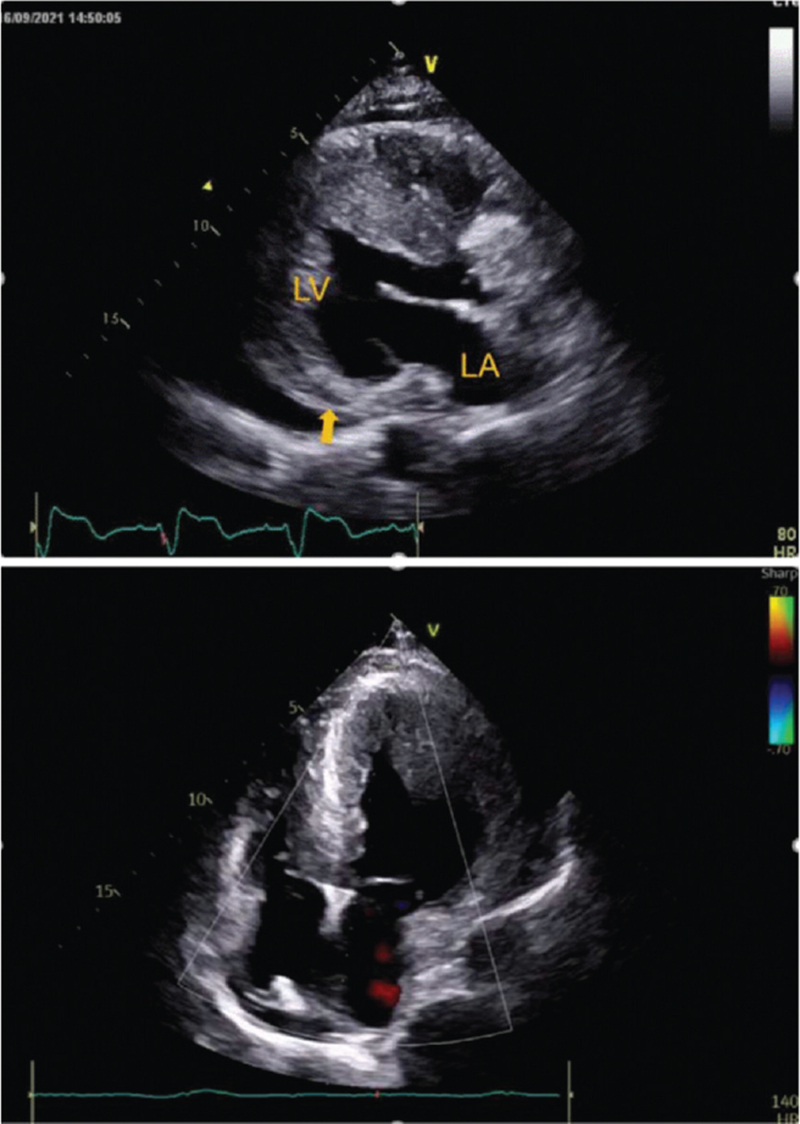
Echocardiogram conducted at our hospital: hypertrophy, ejection fraction 40%, pericardial effusion.

**Figure 4:**
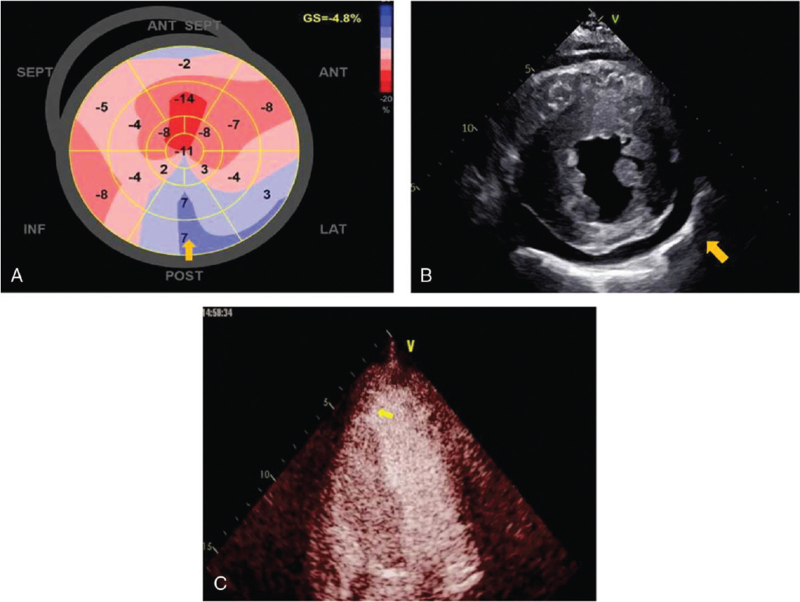
(A and B) Speckle tracking two-dimensional echocardiography strain analysis technology: left ventricular thrombosis, decreased movement of the inferior and posterior walls, and the basal segment of the posterior wall was locally fibrotic. (C) Myocardial contrast echocardiography: left ventricular thrombosis.

**Figure 5:**
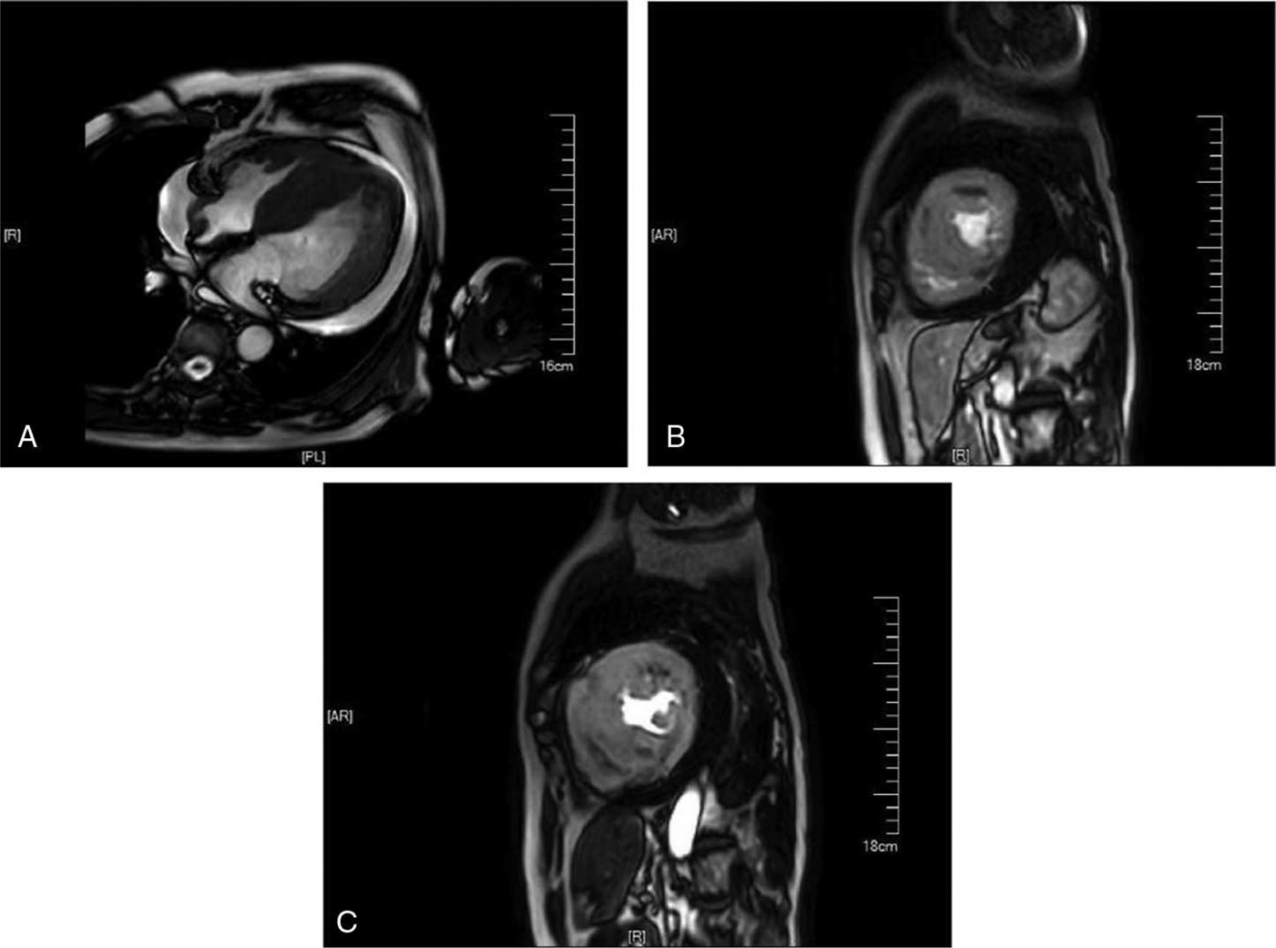
Cardiovascular magnetic resonance imaging: biventricular uneven hypertrophy, which was considered metabolic cardiomyopathy, with diffuse fibrosis of biventricular walls, apical thrombosis, and ischemic cardiomyopathy in the basal segment of the left ventricular lateral wall and left ventricular anterior wall.

**Figure 6:**
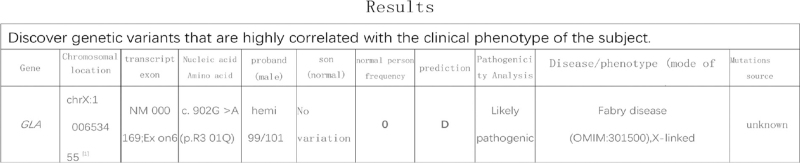
Subsequent genetic testing: missense mutation (c.902G>A) inexon 6 of the *GLA* gene,^[1]^ which induce p.R301Q (p.Arg301Gln).

A 72-year-old man was admitted to the local hospital with intermittent chest pain and heart palpitations; no dyspnea or edema. He had hearing loss for 10 years and wore hearing aids. There was no history of hypertension or diabetes, and no family history of cardiomyopathy, arrhythmia, or sudden cardiac death. A physical examination showed that he was hemodynamically stable and an electrocardiogram (ECG) showed ventricular tachycardia (Fig. 1A). After intravenous amiodarone, his ECG showed sinus rhythm, 72/min, left axis deviation on ECG, left anterior branch block, and intraventricular block. The single obvious abnormality was an ST segment depression with a preterminal negative T wave in leads I and aVL (Fig. 1B). Relevant laboratory test results 12 hours after admission included elevated serum concentrations of myoglobin (86.3 ng/mL, normal range 28–72 ng/mL), cardiac troponin (CTnI) (2.85 ng/mL, normal <0.034 ng/mL), creatine kinase-MB (64.6 U/L, normal range 0–24 U/L), and N-terminal pro B-type natriuretic peptide (7170 ng/mL, normal 300–900 ng/mL). One day later, serum myo-inositol decreased to a normal level (50.3 ng/mL), CTnI decreased to 1.18 ng/mL, and creatine kinase-MB to 29 U/L. Coronary angiography revealed regular findings (Fig. 2). Two weeks later, he still had symptoms of palpitation, and his ECG showed paroxysmal ventricular tachycardia; therefore, he was transferred to our hospital. CTnI decreased to a normal level. Echocardiogram conducted at our hospital revealed hypertrophy, ejection fraction 40%, pericardial effusion (Fig. 3). Speckle tracking two-dimensional echocardiography strain analysis technology confirmed left ventricular thrombosis, and also revealed decreased movement of the inferior and posterior walls, the basal segment of the posterior wall was locally fibrotic (Fig. 4A and B). Further, myocardial contrast echocardiography confirmed left ventricular thrombosis (Fig. 4C). Cardiovascular magnetic resonance imaging indicated biventricular uneven hypertrophy, which was considered metabolic cardiomyopathy, with diffuse fibrosis of biventricular walls, apical thrombosis, and ischemic cardiomyopathy in the basal segment of the left ventricular lateral wall and left ventricular anterior wall (Fig. 5). Serum alpha-galactosidase concentration was 0.7 nmol/h/mgPr (normal range, 29.0–64.4 nmol/h/mgPr). Subsequent genetic testing revealed that he was hemizygous for a previously reported missense mutation (c.902G>A) inexon 6 of the *GLA* gene,^[1]^ which induce p.R301Q (p.Arg301Gln), confirming a diagnosis of FD (Fig. 6). Myocardial biopsy was not performed. Orally administered drugs included rivaroxaban, sacubitril valsartan, beta blockers, dapagliflozin, and mineralocorticoid receptor antagonist. Cardiac resynchronization therapy with an implanted defibrillator was implemented to prevent sudden death. For economic reasons, the patient did not receive enzyme replacement therapy (ERT). At present, he is still in follow-up and there have been no adverse events.

## 3 Discussion

FD is a hereditary disorder linked to the X chromosome, meaning that men have a severe form of the disease and transmit it to all of their daughters, but not to their sons. Fabry disease can manifest as an earlier onset form that effects multiple organ systems or a later onset form that usually dominated by single organ system, which is often the heart.^[5]^ The case presented here appears to fall into the latter category. The main clinical manifestations of cardiac type FD are myocardial hypertrophy; however, the first symptoms in our patient were myocardial infarction, accompanied by left ventricular thrombosis and pericardial effusion.

The patient had typical chest pain and dynamic myocardial enzyme changes and, combined with the results of coronary angiography and echocardiography, this led to a diagnosis of myocardial infarction with non-obstructive coronary artery disease. FD cardiomyopathy include myocyte hypertrophy and fibrosis, which cause raised coronary vascular resistance and increased myocardial oxygen demand.^[6]^

Patients with FD have risk of clinically relevant thromboembolic events, which occur at an incidence rate of 15%, including arterial thrombosis, deep vein thrombosis, and pulmonary embolism, which can be aggravated by concurrent factor V Leiden.^[7,8]^ However, LVT is rarely observed in FD.^[9]^ LVT is a common complication of myocardial infarction and can also occur because of hypertrophic cardiomyopathies, nonischemic dilated cardiomyopathies, and malignancies, among other causes.^[10]^ We speculate that the reasons underlying LVT in our case were related to acute myocardial infarction, low ventricular ejection fraction, and hypercoagulable state, all caused by FD.

Pericardial effusion is a rare manifestation of FD.^[11]^ The presence of lysosomal Gb3 and related globotriaosylsphingosine in pericardial fluid from patients with FD and elevated IL-6 and IL-18 levels in pericardial fluid and plasma, are markers of FD-associated cardiomyopathy severity. Our patient had moderate hemodynamically stable effusion; therefore, we did not conduct pericardiocentesis.

Birket et al demonstrated enhanced sodium and calcium channel function that resulted in higher and shorter spontaneous action potentials in FD cardiomyocytes derived from induced pluripotent stem cells.^[12]^ These findings suggest that stored glycosphingolipids may alter ion channel expression and/or cell membrane trafficking, altering the electrical properties of cardiomyocytes. Namdar et al proposed increased conduction velocity in atrial and ventricular myocardium as possible causes of electrocardiographic abnormalities in FD, including a short PR interval, with no evidence of an accessory pathway.^[13]^ Hence, both tachycardia and conduction block can occur in patients with FD. Our case had a PR interval of <120 ms, and non-sustained ventricular tachycardiadue to enhanced sodium and calcium channel function. Further, the patient had an intraventricular conduction block, possibly related to cardiac fibrosis.

Male patients with late-onset FD have higher residual a-Gal A activity than those with classic FD, although values are far below normal, as in the case described here. Our male patient of 72 years old with only heart involvement in FD is very rare. Patients with FD and type B or AB blood groups often have early onset severe disease, because these two blood groups also carry two other sugar sphingolipids (B and B1 sugar sphingolipids). When α-galactosidase A is deficient, molecular terminal α-galactose residues cannot dissociate, and B and B1 sugar sphingolipids are also deposited in organs and tissues. Since patients with blood groups B or AB have more sugar sphingolipid deposition than those with blood groups O or A, their condition is more serious^[14]^; however, the case presented here has blood group AB, so it is difficult to explain his relatively mild clinical manifestations.

ERT has been shown to benefit various Fabry-related symptoms and organ manifestations.^[15]^ Several studies have shown that ERT provides optimal benefit when it is started at an early disease stage, before extensive fibrosis or other irreversible tissue damage occurs.^[16–19]^

Here we report a patient with late-onset FD presenting with acute myocardial infarction, left ventricular thrombosis, and pericardial effusion. This is a 72-year-old man, only heart involved, and have no symptom of heart failure. That means the process of FD maybe slower than young patient. Although he did not receive ERT, we think maybe he will get benefit from cardiac resynchronization therapy with an implanted defibrillator.

## Acknowledgments

The authors would like to thank the patient and her family for granting us their permission to publish this case report.

## Author contributions

**Following-up:** Hang Li.

**Investigation:** Xiaocong Wang.

**Supervision:** Hui Xu.

**Visualization:** Xiaocong Wang.

**Writing – original draft preparation, reviewing:** Shanshan Zhou.

**Writing – reviewing & editing:** Liping Zhang.
